# Deep learning-based risk stratification of ductal carcinoma *in situ* using mammography and abbreviated breast magnetic resonance imaging

**DOI:** 10.3389/fonc.2025.1587882

**Published:** 2025-06-24

**Authors:** Tingfeng Zhang, Tingting Cui, Zhenjie Cao, Jintao Hu, Jie Ma

**Affiliations:** ^1^ Division of Breast Surgery, Department of General Surgery, Shenzhen People’s Hospital (The Second Clinical Medical College, Jinan University; The First Affiliated Hospital, Southern University of Science and Technology), Shenzhen, Guangdong, China; ^2^ Key Laboratory of Biomedical Engineering of Guangdong Province, South China University of Technology, Guangzhou, China; ^3^ Shenzhen International Graduate School, Tsinghua University, Shenzhen, Guangdong, China; ^4^ Department of Pathology, The Second Affiliated Hospital of Jinan University, Shenzhen People’s Hospital, Shenzhen, China; ^5^ Department of Anatomical and Cellular Pathology, Prince of Wales Hospital, The Chinese University of Hong Kong, Hong Kong, Hong Kong SAR, China; ^6^ Department of Radiology, Shenzhen People’s Hospital, The Second Clinical Medical College of Jinan University, Shenzhen, China

**Keywords:** abbreviated magnetic resonance imaging, deep learning, prognosis, ductal carcinoma *in situ*, mammography

## Abstract

**Background:**

Current management of ductal carcinoma in situ lacks robust risk stratification tools, leading to universal surgical and radiotherapy interventions despite heterogeneous progression risks. Optimizing therapeutic balance remains a critical unmet clinical need.

**Materials and methods:**

We retrospectively analyzed two patient cohorts. The first included 173 cases with BI-RADS category 3 or higher findings, used to compare the diagnostic accuracy of four abbreviated MRI protocols against the full diagnostic MRI. The second cohort involved 210 patients who had both mammography and abbreviated MRI. We developed two separate predictive models—one for pure ductal carcinoma in situ and another for invasive ductal carcinoma with associated ductal carcinoma in situ—by integrating clinical, imaging, and pathological features. Deep learning and natural language processing techniques were used to extract relevant features, and model performance was assessed using bootstrap validation.

**Results:**

Abbreviated Magnetic Resonance Imaging protocols demonstrated similar diagnostic accuracy to the full protocol (P > 0.05), offering a faster yet effective imaging option. The pure group incorporated features like nuclear grade, calcification morphology, and lesion size, achieving an Area Under the Curve of 0.905, with 86.8% accuracy and an F1 score of 0.853. The model for invasive cases incorporated features Ki-67 status, lymph vascular invasion, and enhancement patterns, achieved an Area Under the Curve of 0.880, with 86.2% accuracy and an F1 score of 0.834. Both models showed good calibration and clinical utility, as confirmed by bootstrap resampling and decision curve analysis.

**Conclusion:**

Deep Learning-driven multimodal models enable precise ductal carcinoma *in situ* risk stratification, addressing overtreatment challenges. abbreviated Magnetic Resonance Imaging achieves diagnostic parity with full diagnostic protocol, positioning Magnetic Resonance Imaging as a viable ductal carcinoma *in situ* screening modality.

## 1 Introduction

Ductal carcinoma *in situ* (DCIS) is an early non-invasive lesion of breast cancer, accounting for approximately 18.6% of all newly diagnosed breast cancers (1). DCIS is generally considered a direct precursor to invasive ductal carcinoma (IDC), with approximately 25%-60% of untreated DCIS cases progressing to IDC over a follow-up period of 9–24 years (2). Due to the difficulty in distinguishing high-risk from low-risk DCIS, nearly all DCIS patients undergo either mastectomy or breast-conserving surgery, followed by radiotherapy or endocrine treatment (3). However, treatment of DCIS has not significantly reduced the incidence of invasive breast cancer, suggesting that some DCIS cases may not progress to invasive cancer (4, 5). Therefore, balancing overtreatment and undertreatment of DCIS has become a key clinical concern (6). Mammography is the conventional method for diagnosing DCIS (7), but its diagnostic efficacy is significantly affected by glandular density and tissue overlap (8, 9). Therefore, MRI, which is not influenced by glandular density and has higher sensitivity, is gradually being used as an adjunctive imaging tool for DCIS (10–13).

Artificial intelligence (AI), particularly deep learning (DL) technology, has shown great potential in the detection, diagnosis, and risk assessment of DCIS (14–18). AI algorithms based on mammography have been used to automatically identify calcified lesions, increasing the detection rate of DCIS and reducing the workload of radiologists (16, 19, 20). Furthermore, combining magnetic resonance imaging (MRI) with AI analysis can effectively overcome the limitations of traditional imaging caused by breast density, improving the sensitivity and diagnostic consistency of DCIS (21). However, due to the high time cost of MRI, it is not suitable for routine screening. Therefore, abbreviated breast MRI (Ab-MRI), which significantly reduces examination time, has gradually gained clinical acceptance (22). Nevertheless, current research on Ab-MRI mainly focuses on high-risk breast cancer populations and breast cancer screening, with limited studies using AI to analyze mammography and Ab-MRI image features for DCIS.

This study will combine clinical information with mammography and Ab-MRI image features to develop a prognostic risk prediction model for breast DCIS based on deep learning and multimodal imaging. It will also explore the value of Ab-MRI in the diagnosis and risk stratification of breast DCIS.

## 2 Materials and methods

### 2.1 Data set

**Figure 1:**
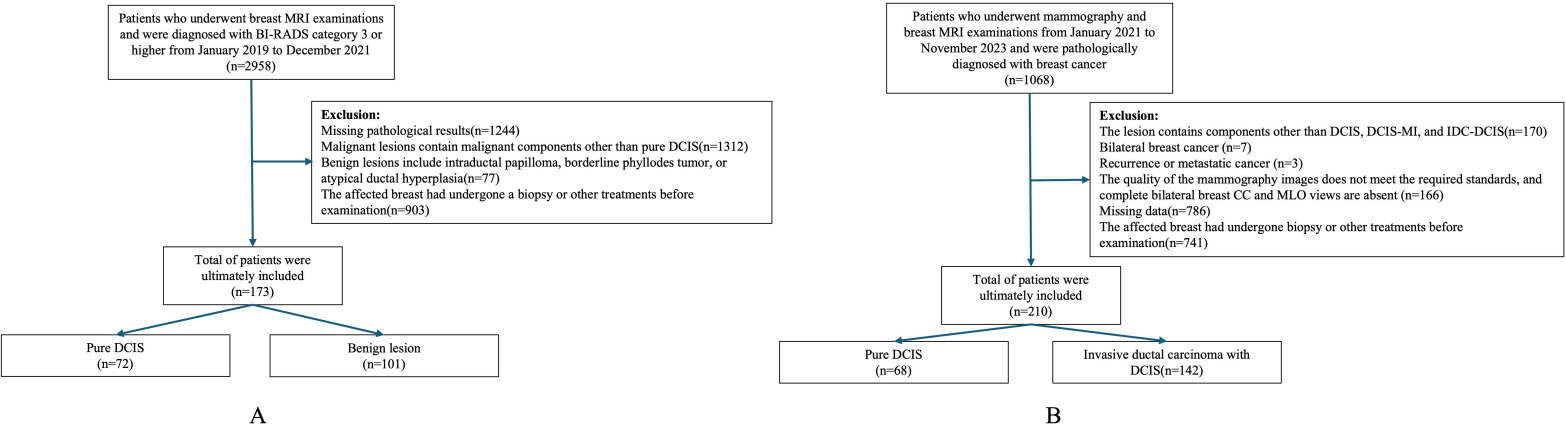
Data collection.

In Dataset One, We retrospectively collected data on 173 patients who underwent breast MRI examinations at our institution between January 2019 and December 2021 and were diagnosed as BI-RADS category 3 or higher. Among them, 72 cases were pure DCIS, and 101 cases were benign lesions. All patients were female, aged 23 to 77 years, with a median age of 46.4 ± 9.3 years. Inclusion criteria (1): Patients with pure DCIS who underwent breast-conserving surgery or total mastectomy after MRI examination (2). Patients with benign lesions who underwent at least core needle biopsy after MRI and had complete postoperative pathological data. Exclusion criteria (1): Malignant lesions containing components other than pure DCIS (2). Benign lesions associated with intraductal papilloma, borderline phyllodes tumor, or atypical ductal hyperplasia (3). Patients who underwent biopsy or other treatments on the affected breast before imaging examination (**Figure 1A**).

In Dataset Two, we collected data from 210 patients who underwent both mammography and breast MRI examinations at our institution between January 2021 and November 2023 and were pathologically confirmed to have breast cancer. Among them, 37 cases were pure DCIS, 31 cases were DCIS with microinvasion (DCIS-MI), and 142 cases were invasive ductal carcinoma with DCIS (IDC-DCIS). All patients were female, aged 29 to 71 years, with a median age of 49.0 ± 9.8 years. Given the similarity in clinical presentation and management strategies between pure DCIS and DCIS-MI, these two groups were combined for analysis. IDC-DCIS cases were characterized by predominant DCIS lesions with accompanying invasive components. Inclusion criteria (1): Pathologically confirmed DCIS-containing lesions (2); Underwent breast-conserving surgery or total mastectomy after mammography (3); Had complete clinical, imaging, pathological, and immunohistochemistry (IHC) data. Exclusion criteria (1): Lesions containing components other than DCIS, DCIS-MI, or IDC-DCIS (2); Bilateral breast cancer (3); Recurrent or metastatic breast cancer (4); Poor imaging quality or missing complete craniocaudal (CC) and mediolateral oblique (MLO) mammographic views for both breasts (5); Prior biopsy or other treatments on the affected breast before imaging examination (**Figure 1B**).

### 2.2 Examination methods

**Figure 2:**
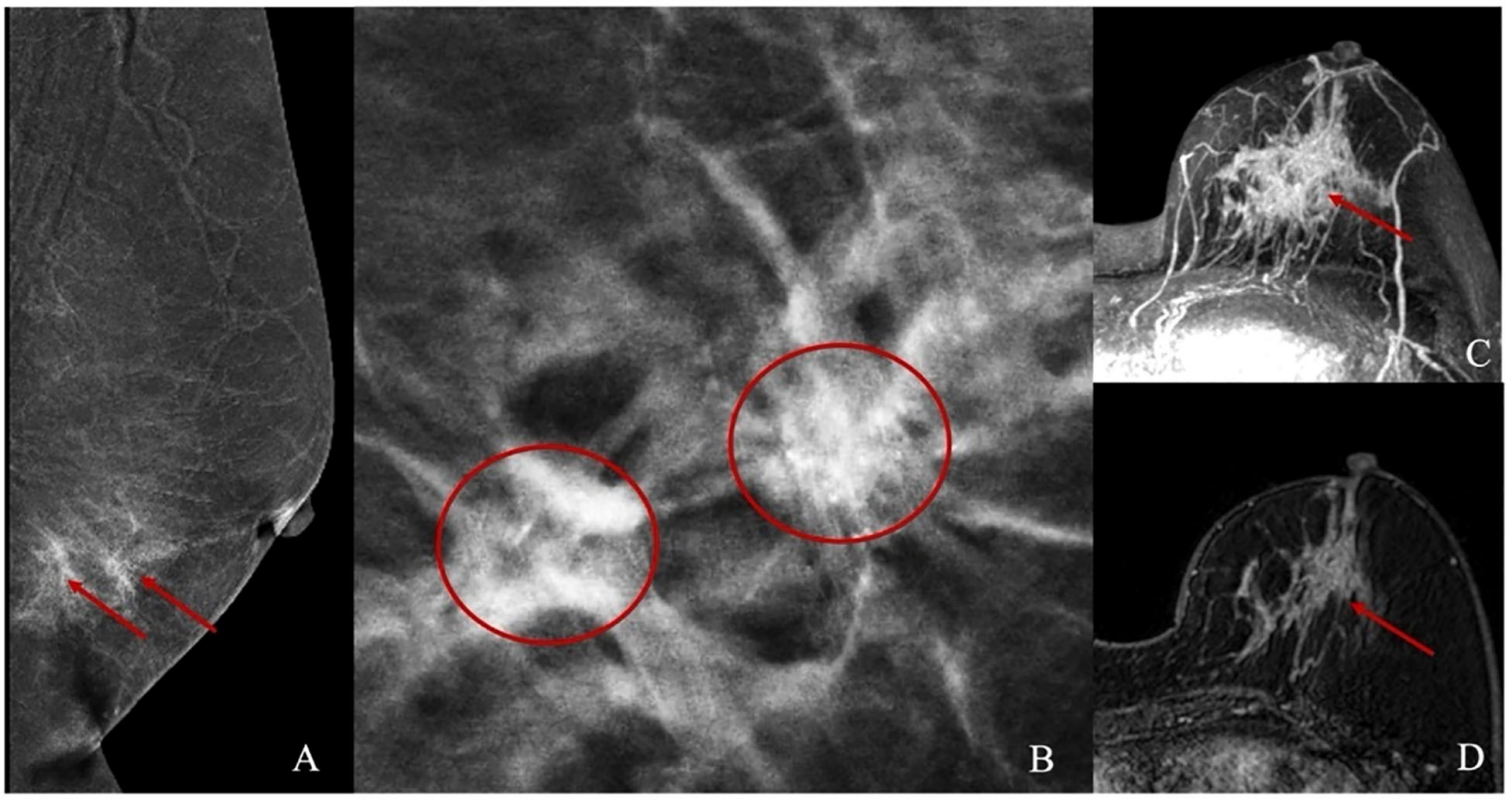
Illustrations of Mammography and Abbreviated Breast MRI. **(A, B)** present the mammographic images of a patient with non-mass enhancement and the corresponding calcification location. **(C, D)** depict the non-mass enhancement characteristics observed in the Abbreviated Breast MRI images.

Breast mammography examinations were performed the following equipment: Siemens Mammomat Inspiration digital mammography system, GIOTTO IMAGE MD dual flat-panel digital mammography system, GE Senographe Pristina digital mammography system, and Hologic Selenia Dimensions mammography system. Standard CC and MLO views were acquired using a fully automatic compression system and automatic exposure control mode to optimize image quality (**Figures 2A, B**).

MRI exams were conducted on Siemens Skyra 3.0T and Avanto 1.5T scanners with a dedicated breast coil, with patients positioned prone and both breasts naturally hanging. Unenhanced sequences included: T2-weighted imaging with fat suppression (T2WI-FS): Axial imaging with repetition time (TR) 3400/5000ms, echo time (TE) 54/58ms, slice thickness 4 mm, and slice gap 0.8/0.4 mm. Diffusion-weighted imaging (DWI): TR 5700/6400ms, TE 59/97 ms, slice thickness 4 mm, and slice gap 0.8/2 mm. Contrast-enhanced imaging was done 90 seconds after unenhanced imaging, with the first postcontrast phase using the following parameters: TR: 4.66/5.16ms. TE: 1.68/2.39ms. Slice thickness: 1.6/1.1 mm. Slice gap: 0.32/0.22 mm Contrast agent: Gadobenate dimeglumine, administered at 2.0 ml/s with a dose of 0.2 mmol/kg. Post-processing included maximum intensity projection (MIP) and first postcontrast subtracted (FAST) image reconstruction (**Figures 2C, D**).

### 2.3 Abbreviated breast MRI program screening

Given the unique biological characteristics of malignant tumors, this study incorporated DWI and early post-contrast images into the dynamic imaging protocol, with four imaging combinations designed as follows (1): T2WI-FS + DWI (2), T2WI-FS + MIP (3), DWI + MIP + FAST, and (4) T2WI-FS + DWI + MIP + FAST (12). A blinded reading approach was employed, where two radiologists with over five years of experience in breast MRI diagnosis independently evaluated cases from Dataset One using the four Ab-MRI protocols alongside the full diagnostic protocol (FDP). Each case was classified according to the BI-RADS system, categorizing them as BI-RADS 1, 2, 3 or BI-RADS 4, 5, without access to clinical data or other examination results. In cases of disagreement, a final classification was determined through consensus discussion. To minimize bias from repeated readings, a one-month interval was maintained between assessments for each protocol. Finally, the diagnostic performance of the four Ab-MRI protocols was compared with FDP, and the most effective Ab-MRI protocol was selected for further study.

### 2.4 Radiological feature extraction

In the mammographic image feature analysis, 273 patients were randomly divided into a training set of 162 cases (simple group: 66 cases, invasive group: 96 cases) and a validation set (simple group: 44 cases, invasive group: 67 cases). This study adopted the AI model framework from the research team’s previous work (23) to analyze and extract both imaging and textual features, including breast tissue density, BI-RADS classification, and mammographic features. Building upon this, the MommiNet-v2 model was employed as a multi-view breast mass detection and classification system that simulates the radiologist’s clinical practice of comparing ipsilateral and contralateral views. It takes three-view mammograms along with clinical information (BI-RADS scores and biopsy results) as input. Structural and symmetry features are extracted through IpsiDualNet-v2 and BiDualNet-v2, then fused in an integrated network to generate final predictions. The training process incorporates nipple-based alignment, Focal Loss, and Distance-IoU Loss to enhance detection and classification accuracy. MommiNet-v2 has demonstrated strong performance on both public and real-world datasets, indicating its potential value for clinical implementation. According to the ACR BI-RADS guidelines (24), suspicious calcification shapes in mammography include amorphous, coarse heterogeneous, fine pleomorphic, and linear branching calcifications, while associated signs include skin retraction, nipple inversion, skin thickening, trabecular thickening, and axillary lymphadenopathy.

**Figure 3:**
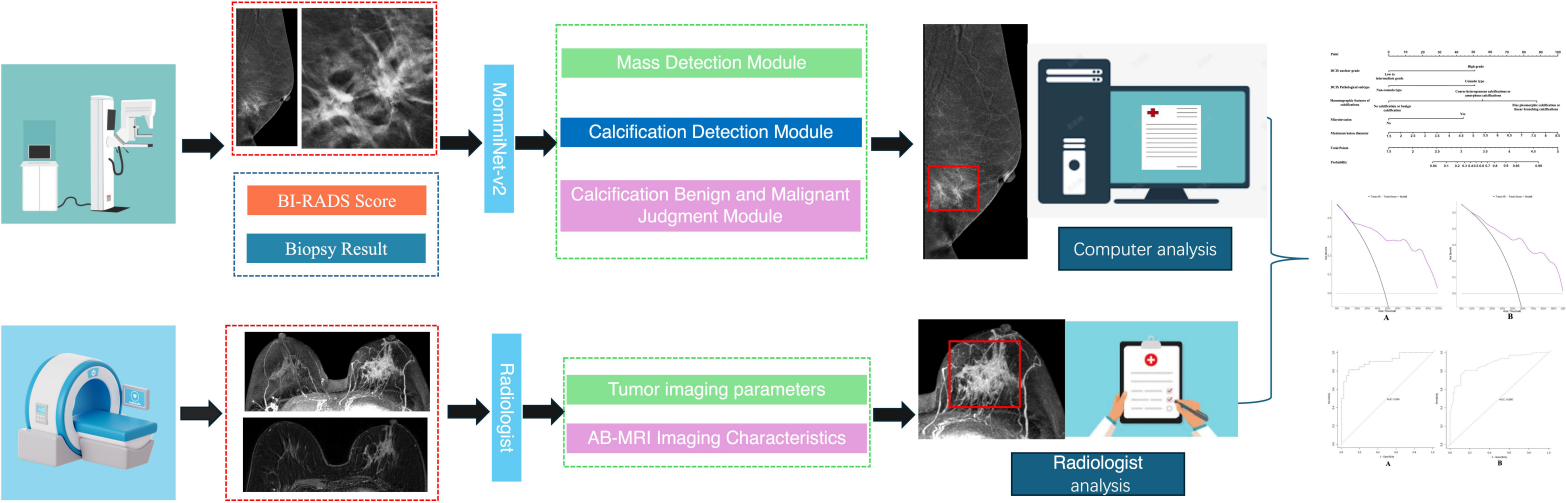
Shows the flow chart of the study.

In the MRI image feature analysis, two radiologists with over five years of experience in breast MRI diagnosis evaluated Dataset Two using the selected optimal Ab-MRI protocol, with prior knowledge of patient clinical information, and recorded the degree of breast parenchymal enhancement, type of lesion enhancement, maximum lesion diameter, apparent diffusion coefficient (ADC) at high b-values, and the presence or absence of clustered ring enhancement within the lesion. If there was any discrepancy between the two radiologists, a final interpretation was reached through discussion. **Figure 3** shows the flow chart of the study.

### 2.5 Pathological feature extraction

In the pathological analysis, the DCIS nuclear grade (low-intermediate/high) and pathological subtype (non-comedo/comedo) were recorded. Additionally, for the pure DCIS group, the presence or absence of microinvasion was noted, while for the invasive group, the proliferation index (Ki67), DCIS component proportion (using 25% as the threshold based on the concept of extensive intraductal component, EIC), and the presence or absence of neural/vascular invasion were also assessed. All specimens underwent IHC staining to evaluate the expression of Estrogen Receptor(ER), Progesterone Receptor(PR), Human Epidermal Growth Factor Receptor 2 (HER2), and Ki67. For patients in the invasive group, if discrepancies in IHC expression levels were observed between IDC and DCIS, the DCIS results were used as the reference. ER and PR positivity were defined as ≥1% nuclear staining. HER2 positivity was defined as 3+ (strong and complete membrane staining in >10% of tumor cells), while HER2 1+/0 was considered negative. For HER2 2+ cases, fluorescence *in situ* hybridization (FISH) was performed to confirm HER2 status, with HER2 gene amplification or FISH positivity indicating HER2 positivity. Ki67-positive cells ≤14% were classified as low proliferative activity, while >14% indicated high proliferative activity (25). All pathological results were independently reviewed by two attending pathologists with over 10 years of experience in breast pathology diagnosis. In cases of discrepancies, a panel of expert pathologists conducted a consensus review to determine the final interpretation.

### 2.6 Prediction model construction

In the pure DCIS group prediction model, a total of eight features were extracted, including Ab-MRI image features, mammography image features, and non-imaging clinical features. These specific features were DCIS nuclear grade, DCIS pathological subtype, Mammographic features of calcifications, Presence of microinvasion, Degree of background parenchymal enhancement, Lesion enhancement pattern (derived from DCE-MRI), Maximum lesion diameter, Apparent diffusion coefficient value of the lesion. In the invasive group prediction model, nine features were extracted across the same three categories. These features included: Nerve/vascular invasion, Ki-67 proliferation index, Mammographic associated findings, Proportion of DCIS component, Degree of background parenchymal enhancement, Lesion enhancement pattern, Maximum lesion diameter, Apparent diffusion coefficient value, Clustered ring-like enhancement within the lesion.

All extracted features were initially subjected to univariate analysis, and those with statistical significance (P < 0.05) were subsequently included in multivariate logistic regression analysis. Features that remained significant in the multivariate analysis (P < 0.05) were identified as independent predictors of prognosis in both the pure DCIS and invasive cohorts.

To assess the performance of different model construction strategies, we used the Akaike Information Criterion (AIC) to evaluate model fit. The final models—the pure DCIS + DCIS-MI prediction model and the IDC-DCIS prediction model—were selected based on achieving the lowest AIC values, ensuring optimal balance between goodness-of-fit and model complexity.

### 2.7 Prognostic evaluation criteria

ER-negative or HER2-positive was selected as the reference standard for poor prognosis in the pure DCIS group (26–28). For the invasive group, the Nottingham Prognosis Index (NPI) (29) was used as the reference standard for poor prognosis, with NPI < 4.4 defined as good prognosis and NPI ≥ 4.4 as poor prognosis.

### 2.8 Statistical analysis

In the Ab-MRI protocol selection, SPSS 26.0 was used for statistical analysis to calculate diagnostic metrics for each MRI protocol, including positive predictive value (PPV), negative predictive value (NPV), sensitivity (SEN), specificity (SPE), and accuracy (ACC). The McNemar test was applied to compare the diagnostic performance of the Ab-MRI protocol with the full diagnostic protocol (FDP), with P < 0.05 indicating statistical significance. For predictive model establishment, R 4.1.2 was used for data organization and statistical analysis. Categorical data were presented as counts (percentages), and Pearson χ² test or Fisher’s exact test was used to compare differences between groups. For continuous data, if normally distributed, mean value was used, and independent t-test was applied for between-group comparisons; if not normally distributed, median value was used, and Mann-Whitney U test was employed for group comparisons. Univariate analysis was used to identify variables with P < 0.05, which were then included in multivariate logistic regression to establish the prediction model. The R “pROC” package was used to plot the ROC curve, and the “rms” package was used to create a nomogram for model visualization, along with calibration curves to assess model calibration. Additionally, the “dcurves” package was used to plot decision curves, and DCA was applied to assess the clinical net benefit of the model. To further investigate the stability of the model and ensure its fit, Bootstrap resampling (1000 iterations) was performed. P < 0.05 indicates statistical significance.

## 3 Results

### 3.1 Cohort characteristics and abbreviated breast MRI protocol

In Dataset One, this study selected 72 cases of pure DCIS and 101 cases of mixed benign lesions to evaluate the diagnostic performance of the Ab-MRI protocol. Among the benign lesions, there were 19 cases of fibroadenoma, 14 cases of fibrocystic breast disease, 3 cases of benign phyllodes tumor, 1 case of ductal adenoma, 1 case of hamartoma, and 1 case of breast tuberculosis, as well as 62 cases with no pathological result but no disease progression after 2 years of follow-up. The diagnostic performance and comparison of the four Ab-MRI protocols with FDP are shown in **Tables 1**, **2**. In terms of scanning time, T2WI-FS was not affected by the device model, with scanning times exceeding 3 minutes; however, its diagnostic value was limited. In contrast, Protocol 3 (DWI, MIP, FAST) had a shorter scanning time and showed higher PPV, NPV, SEN and ACC. When comparing the four Ab-MRI protocols with the FDP, only Protocol 1 showed a significant difference in SEN compared to the FDP (P = 0.020), while no significant differences were found in sensitivity, specificity, or accuracy for the other protocols (P > 0.05). Therefore, the “DWI, MIP, FAST” protocol was selected for further study.

**Table 1:** Examination time and diagnostic performance of different MRI protocols.

Protocol	Scan time3.0T/1.5T	PPV	NPV	SEN	SPE	ACC
T2WI-FS、DWI	8 min 03 s/5 min 59 s	0.527 (42.1%–63.3%)	0.667 (55.2%–77.1%)	54.2% (43.7%–64.3%)	65.3% (55.1%–74.4%)	60.7% (53.2%–67.8%)
T2WI-FS、MIP	5 min 39 s/4 min 49 s	0.666 (54.8%–76.5%)	0.890 (82.3%–93.1%)	86.1% (77.2%–92.0%)	73.2% (64.1%–80.8%)	75.7% (69.0%–81.5%)
DWI、MIP、FAST	5 min 42 s/4 min22 s	0.716 (62.4%–80.0%)	0.894 (83.5%–93.2%)	87.5% (79.3%–92.9%)	73.9% (65.0%–81.3%)	80.3% (74.1%–85.5%)
T2WI-FS、DWI MIP、FAST	9 min 33 s/7 min 29 s	0.702 (60.1%–79.1%)	0.924 (87.6%–95.5%)	91.7% (84.5%–96.0%)	75.3% (67.1%–82.1%)	80.3% (74.1%–85.5%)
FDP	23min 45 s/24 min 17 s	0.831 (72.3%–89.4%)	0.967 (93.1%–98.9%)	95.8% (89.6%–98.5%)	86.1% (78.2%–91.6%)	90.2% (85.0%–93.9%)

**Table 2:** Comparison of diagnostic performance between different Ab-MRI protocols and FDP.

Indicators	Subgroup	T2WI-FS、DWI	T2WI-FS、MIP	DWI、MIP、FAST	T2WI-FS、DWI、MIP、FAST
SEN	x^2^ value	7.838	0.309	0.337	0.004
	P value	0.020	0.069	0.561	0.949
SPE	x^2^ value	0.144	0.773	0.046	0.243
	P value	0.704	0.183	0.830	0.622
ACC	x^2^ value	0.043	0.119	0.290	0.015
	P value	0.385	0.730	0.590	0.903

In Dataset Two, a total of 210 breast cancer patients were included in this study, with 68 patients in the pure DCIS group, of which 32 had poor prognosis and 36 had good prognosis. Among the 37 pure DCIS patients, 12 had poor prognosis and 25 had good prognosis; among the 31 DCIS-MI patients, 20 had poor prognosis and 11 had good prognosis. The invasive group included 142 patients, with 78 having poor prognosis and 64 having good prognosis. The baseline characteristics of patients in both the pure DCIS and invasive groups, as well as the results of univariate analysis, are presented in **Tables 3**, **4**.

**Table 3:** Baseline characteristics and univariate analysis of the pure DCIS group.

Characteristics	Subgroup	Poor prognosis	Good prognosis	X^2^/ t value	P value
Number		32	36		
DCIS nuclear grade	Low to intermediate grade	10(31.2%)	22(61.1%)	6.063	0.014
	high grade	22(68.8%)	14(38.9%)		
DCIS pathological subtype	Non-comedo type	9(28.1%)	21(58.3%)	6.271	0.012
	Comedo type	23(71.9%)	15(41.7%)		
Mammographic features of calcifications	No calcification or benign calcification	5(15.6%)	21(58.3%)	13.654	0.001
	Coarse heterogeneous calcifications or amorphous calcifications	11(34.4%)	8(22.2%)		
	Fine pleomorphic calcification or linear branching calcifications	16(50.0%)	7(19.4%)		
With microinvasion		20(62.5%)	11(30.6%)	6.969	0.008
Degree of background parenchymal enhancement of the breast	Low enhancement	20(62.5%)	21(58.3%)	0.123	0.726
	High enhancement	12(37.5%)	15(41.7%)		
Lesion enhancement pattern	Mass-like enhancement	9(28.1%)	10(27.8%)	0.340	0.844
	Non-mass-like enhancement	16(50.0%)	20(55.6%)		
	Mass with non-mass-like enhancement	7(21.9%)	6(16.7%)		
Maximum lesion diameter		5.69(1.58)	4.66(1.42)	-2.843	0.006
Lesion ADC value		0.86(0.20)	0.87(0.16)	0.271	0.787

ADC, Apparent Diffusion Coefficient.

**Table 4:** Baseline characteristics and univariate analysis of the invasive group.

Characteristics		Poor prognosis	Good prognosis	Z/t/X^2^ value	P value
Number		78	64		
Nerve/vascular invasion		41(52.6%)	16(25.0%)	11.116	0.001
Ki67 status	Low proliferation index High proliferation index	19(24.4%)	31(48.4%)	8.934	0.003
		59(75.6%)	33(51.6%)		
Mammographic associated findings		16(20.5%)	34(53.1%)	16.389	<0.001
Proportion of DCIS component	>25%	19(24.4%)	43(67.2%)	26.215	<0.001
	≤25%	59(75.6%)	21(32.8%)		
Degree of background parenchymal enhancement of the breast	Low enhancement	54(69.2%)	48(75.0%)	0.578	0.447
	High enhancement	24(30.8%)	16(25.0%)		
Lesion enhancement pattern	Mass-like enhancement	30(38.5%)	44(68.8%)	14.665	0.001
	Non-mass-like enhancement	22(28.2%)	13(20.3%)		
	Mass with non-mass-like enhancement	26(33.3%)	7(10.9%)		
Maximum lesion diameter		2.20[1.60,3.10]	2.00[1.30,3.02]	-0.872	0.383
Lesion ADC value		0.73(0.18)	0.73(0.15)	-0.180	0.857
Clustered ring-like enhancement within the lesion		28(35.9%)	40(62.5%)	9.969	0.002

### 3.2 Development of predictive models

Through univariate comparisons between groups, factors with P < 0.05 were selected to construct the predictive models. In the pure DCIS group, 5 features, including DCIS nuclear grade, DCIS pathological subtype, mammographic calcification morphology, microinvasion, and maximum lesion diameter, were used to construct the predictive model, with an AIC value of 68. In the invasive group, 6 features, including neural/vascular invasion, Ki67 status, DCIS component proportion, mammographic associated findings, lesion enhancement type, and clustered ring enhancement within the lesion, were used to construct the predictive model, with an AIC value of 139.

**Figure 4:**
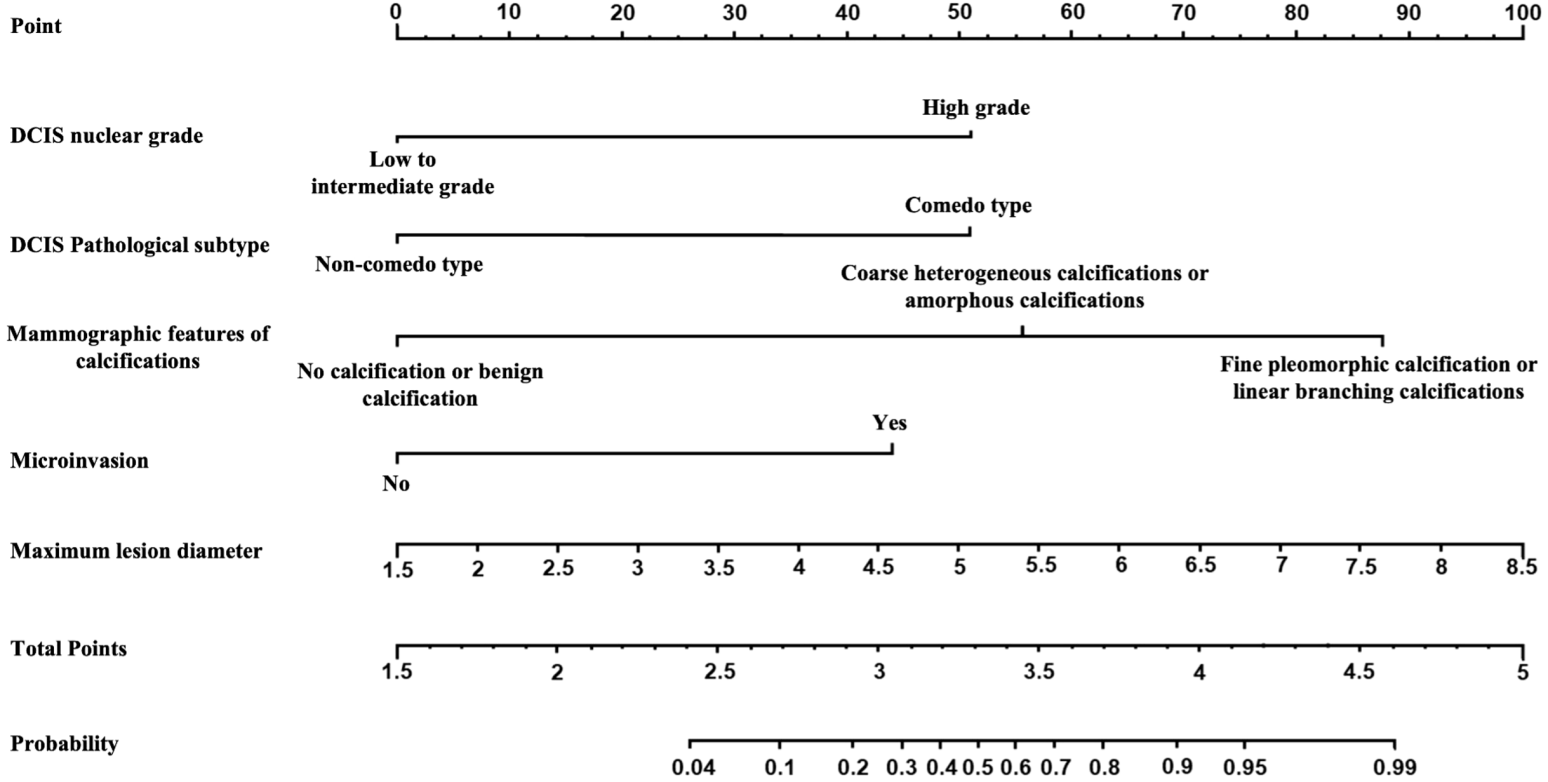
Nomogram of the predictive model for the pure DCIS group.

**Figure 5:**
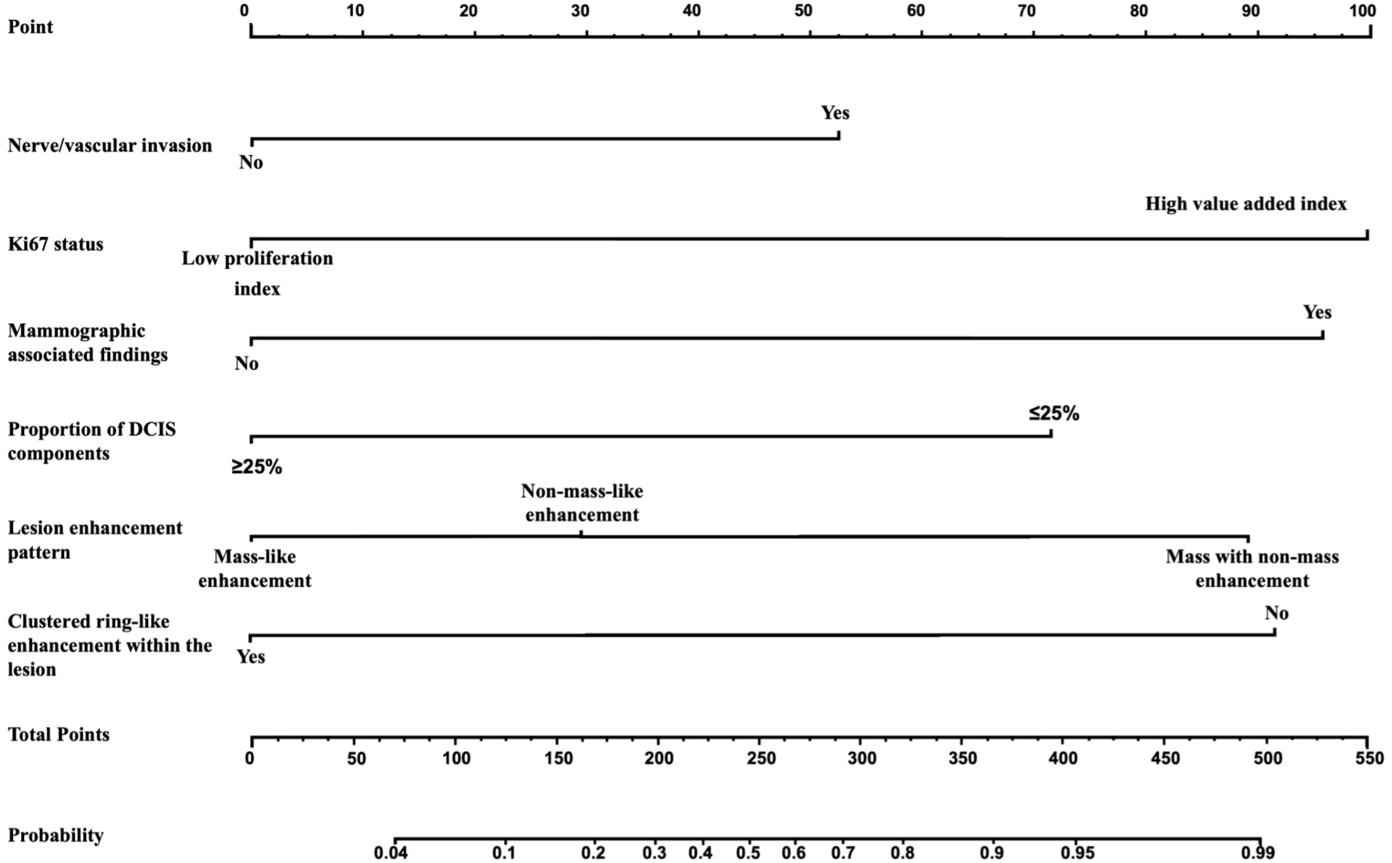
Nomogram of the predictive model for the invasive group.

The multivariate analysis results for the pure DCIS group are shown in **Table 5**. DCIS nuclear grade, DCIS pathological subtype, mammographic calcification morphology, microinvasion, and maximum lesion diameter were identified as independent predictive factors for poor prognosis in the pure DCIS group. The nomogram for this predictive model is shown in **Figure 4**. The multivariate analysis results for the invasive group are shown in **Table 6**. Neural/vascular invasion, Ki67 status, mammographic associated findings, DCIS component proportion, lesion enhancement type, and clustered ring enhancement within the lesion were identified as independent predictive factors for poor prognosis in the invasive group. Among these, neural/vascular invasion, Ki67 status, mammographic associated findings, and lesion enhancement type had Odds Ratio(OR) values greater than 1, indicating these are risk factors, while DCIS component proportion and clustered ring enhancement had OR values less than 1, indicating these are protective factors. The nomogram for this predictive model is shown in **Figure 5**.

**Table 5:** Multivariate logistic regression analysis to predict the prognosis of the pure DCIS group.

Characteristics	Coefficient	St. error	Z value	P value	OR	95%CI
DCIS nuclear grade	1.817	0.763	2.382	0.017	6.156	1.380~27.473
DCIS pathological subtype	1.811	0.734	2.465	0.014	6.114	1.449~25.793
Mammographic features of calcifications						
Coarse heterogeneous. calcifications or amorphous calcifications	1.976	0.905	2.183	0.029	7.215	1.224~42.535
Fine pleomorphic. calcification or linear branching calcifications	3.112	0.975	3.191	0.001	22.463	3.322~151.907
With microinvasion	1.573	0.726	2.165	0.030	4.819	1.161~20.005
Maximum lesion diameter	0.508	0.248	2.046	0.041	1.662	1.021~2.705

St. Error, Standard Error; OR, odds ratio; CI, confidence Interval.

**Table 6:** Multivariate logistic regression analysis to predict the prognosis of the invasive group.

Characteristics	Coefficient	St. error	Z value	P value	OR	95%CI
Nerve/vascular invasion	0.962	0.483	1.991	0.047	2.617	1.015~6.751
Ki67 status	1.831	0.522	3.506	0.000	6.241	2.242~17.374
Mammographic associated findings	1.753	0.485	3.611	0.000	5.770	2.229~14.940
Proportion of DCIS component	-1.308	0.478	-2.738	0.006	0.270	0.106~0.690
Lesion enhancement pattern						
Non-mass-like enhancement	0.539	0.543	0.992	0.321	1.714	0.591~4.969
Mass with non-mass-like enhancement	1.635	0.632	2.588	0.010	5.130	1.487~17.698
Clustered ring-like enhancement within the lesion	-1.676	0.500	-3.353	0.001	0.187	0.070~0.498

St. Error, Standard Error; OR, odds ratio; CI, confidence Interval.

### 3.3 Evaluation of predictive model performance

**Figure 6:**
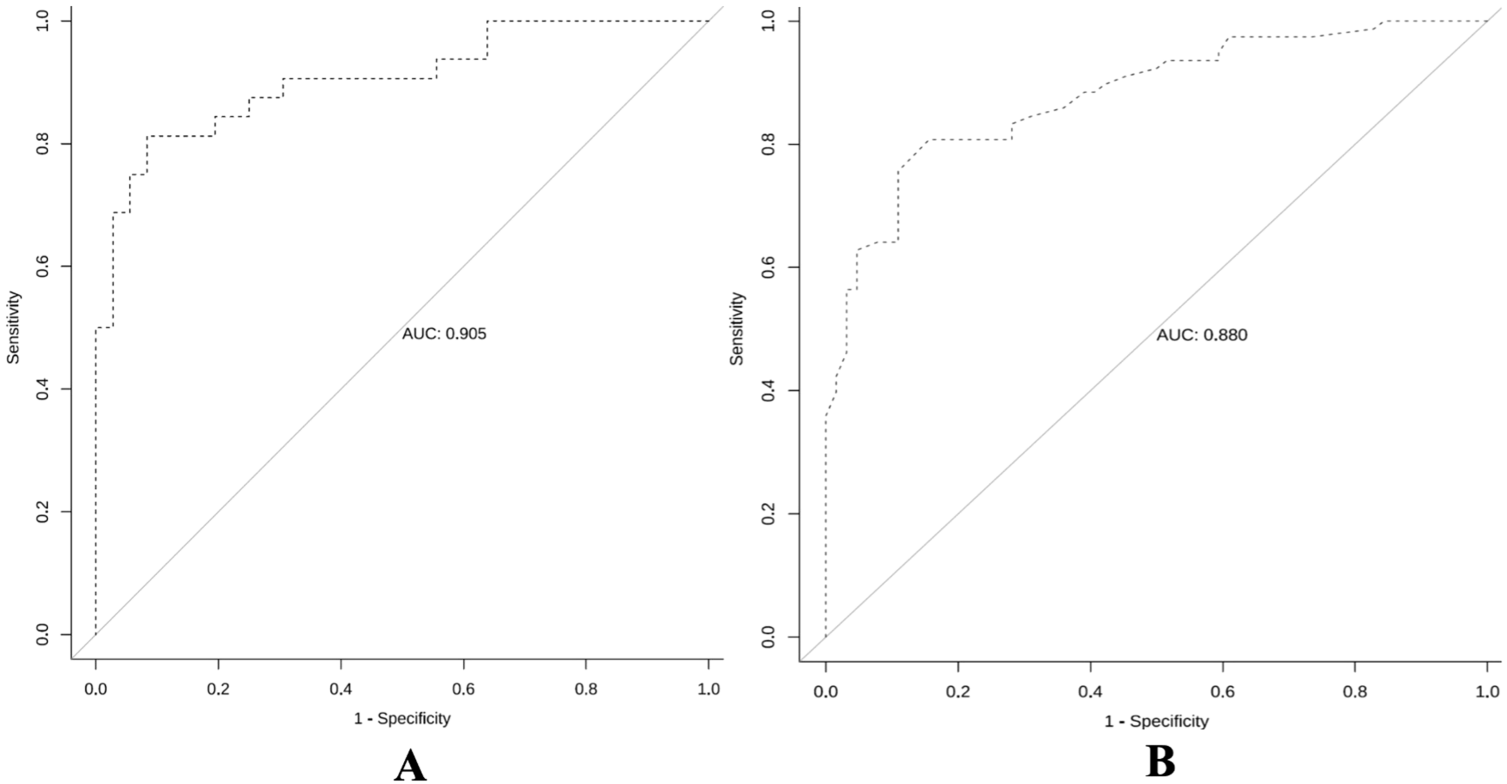
Receiver operating characteristic curves of the predictive models for the pure DCIS group **(A)** and the invasive group **(B)**.

ROC curve analysis (**Figures 6A, B**) showed that the pure DCIS group model for predicting the prognosis of pure DCIS + DCIS-MI had an AUC of 0.905 (95% CI 0.833–0.978). When the cutoff value was T = 0.642, the Youden index was maximized at 0.729, with a specificity of 91.7%(95%CI 82.4–97.5) and a sensitivity of 81.3%(95%CI 70.2–89.8). The corresponding prediction model achieved an accuracy of 86.76%(95%CI 82.1–90.8) and an F1 score of 0.853. The invasive group model for predicting the prognosis of IDC-DCIS had an AUC of 0.880 (95% CI 0.825–0.935). When the cutoff value was T = 0.573, the Youden index was maximized at 0.651, with a specificity of 84.4%(95%CI 75.9–91.2) and a sensitivity of 80.8%(95%CI 71.6–88.3). This model achieved an accuracy of 86.2%(95%CI 81.5–90.1) and an F1 score of 0.834.

To further validate the stability of the model, an internal validation using the Bootstrap resampling method was performed with 1000 repetitions, and the mean AUC and 95% CI of the ROC curve were calculated. The results showed that the mean AUC for the pure DCIS group model was 0.906 (95% CI 0.801–0.957), and the mean AUC for the invasive group model was 0.879 (95% CI 0.817–0.927).

**Figure 7:**
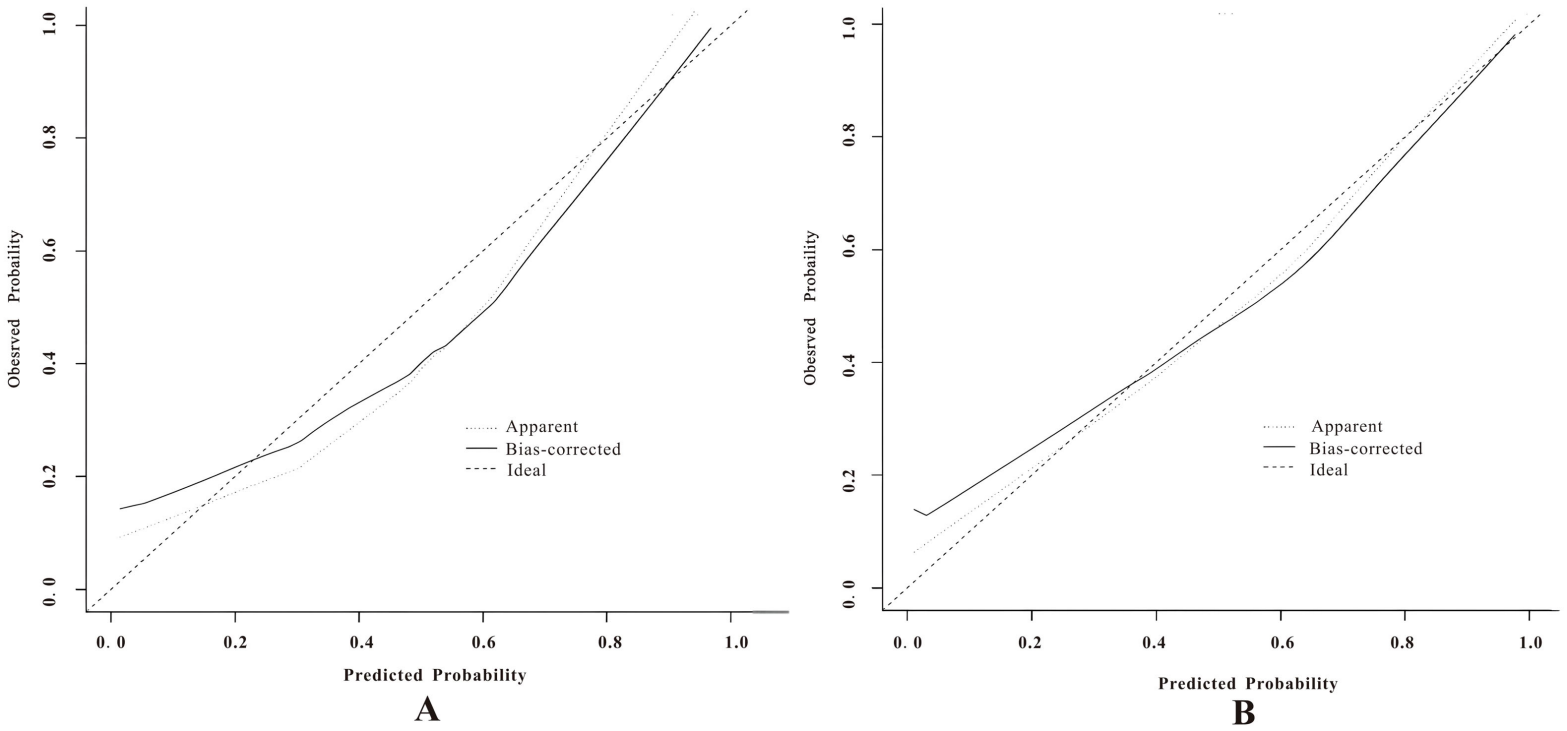
Calibration curves of the predictive models for the pure DCIS group **(A)** and the invasive group **(B)**.

**Figure 8:**
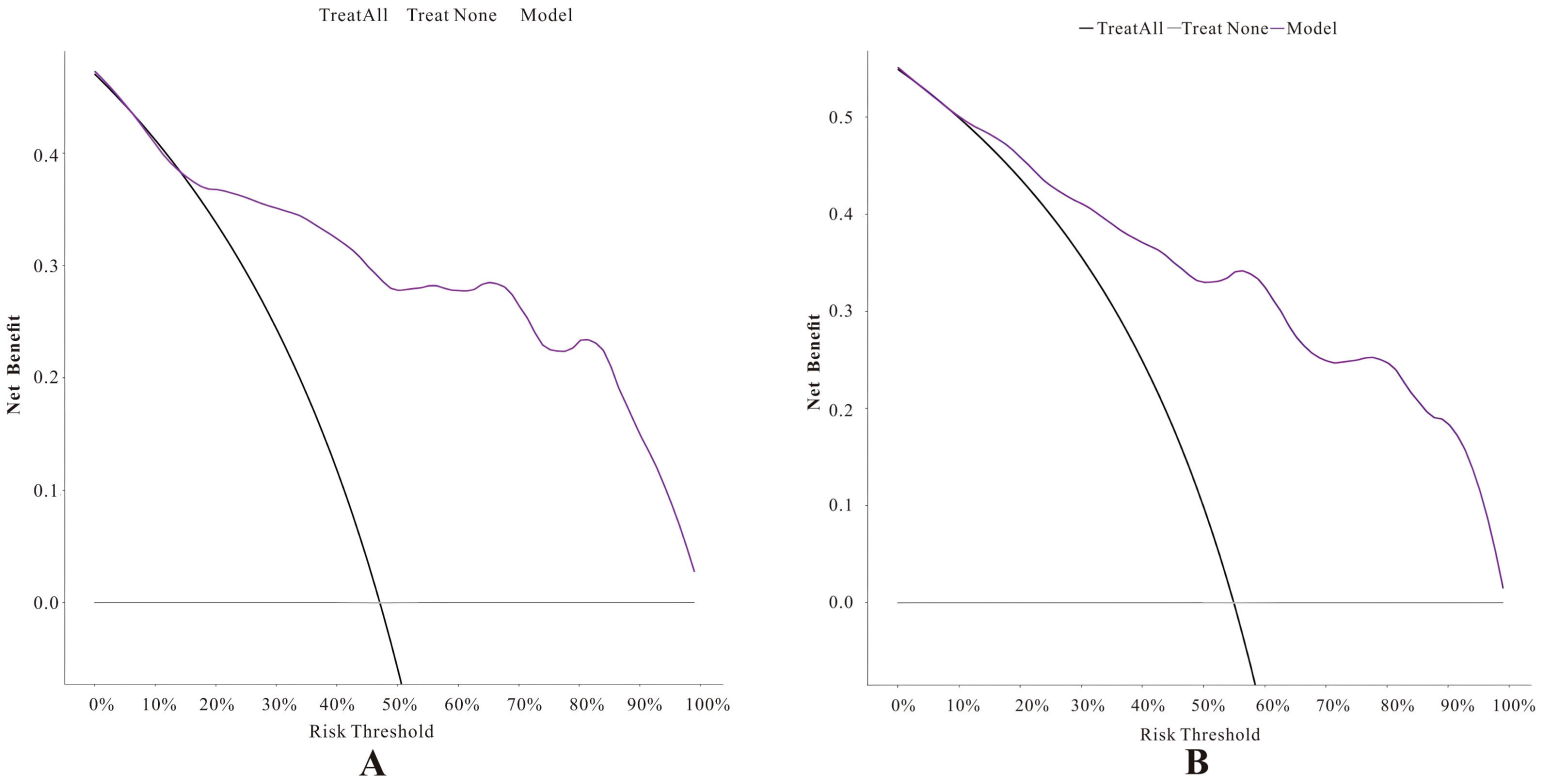
Decision curves of the predictive models for the pure DCIS group **(A)** and the invasive group **(B)**.

In the calibration curve analysis (**Figures 7A, B**), the X-axis represents the predicted risk probability, and the Y-axis represents the actual risk probability. The deviation correction curves of both prediction models fit well with the 45° ideal curve, indicating that the predicted malignant risk probability closely matches the actual malignant risk probability. According to the Hosmer and Lemeshow test, the P value for the pure DCIS model was 0.523, and the P value for the invasive group model was 0.127, both greater than 0.05, suggesting that the model fit is good. Decision curve analysis (**Figures 8A, B**) showed that the prediction model curves for both the pure DCIS group and the invasive group were significantly better than the two extreme lines. When the risk threshold was greater than 0.15 for the pure DCIS group and greater than 0.10 for the invasive group, net benefit was achieved, indicating that the model is beneficial for clinical decision-making.

This study compared the diagnostic performance of four Ab-MRI protocols with the full MRI protocol for DCIS. The DWI, MIP, and FAST protocols were selected for image feature extraction. In the pure DCIS group, the maximum lesion diameter was included, while in the invasive group, the lesion enhancement type and clustered ring enhancement within the lesion were incorporated. The first section’s predictive factors were combined to establish a multimodal prediction model. The results showed that the diagnostic performance of the Ab-MRI protocol for DCIS was not significantly different from the full MRI protocol, and the examination time was notably shorter. Additionally, the Ab-MRI protocol effectively performed risk stratification for DCIS at different pathological stages, with the established prediction model demonstrating good performance in ROC curve analysis, calibration curve analysis, and decision curve analysis.

## 4 Discussion

The extent and distribution of DCIS lesions are difficult to assess, posing a significant challenge for clinicians and patients in determining the appropriate surgical treatment. In the context of approximately 20% of DCIS cases developing invasive breast cancer in the same breast after treatment (30, 31). Therefore, accurate risk stratification can help guide the precise extent of surgery for patients who wish to preserve their breast (32, 33). We evaluated the diagnostic performance of different imaging features by two datasets, employing Ab-MRI protocols including DWI, MIP, and FAST. Our results demonstrate that the model effectively stratifies the risk of DCIS at different pathological stages and shows good performance in ROC, calibration, and decision curve analyses, providing accurate prognostic predictions for clinical use.

DCIS is a localized lesion, it exhibits considerable biological heterogeneity across patients, and some cases may progress to invasive breast cancer. Therefore, accurate diagnosis is essential for guiding appropriate treatment decisions. Mammography remains the primary screening tool for DCIS due to its ability to detect microcalcifications. However, its diagnostic performance is limited by breast density, which can lead to underdiagnosis or misclassification, particularly in non-calcified lesions (34, 35).This limitation may result in incomplete excision during breast-conserving surgery, thereby increasing the risk of recurrence and sometimes necessitating wider surgical resection. In contrast, MRI offers superior spatial resolution and contrast-enhanced imaging capabilities, enabling earlier and more accurate detection of DCIS and holding promise for improved long-term patient outcomes (36, 37). Furthermore, MRI has demonstrated higher sensitivity in detecting additional malignancies, with a detection rate of 87.9% compared to 63.6% for mammography, providing a more comprehensive assessment of disease burden (38).

However, despite its superior diagnostic capabilities in DCIS detection, MRI is not suitable for routine screening due to its high cost and lengthy examination time. As a potential alternative, Ab-MRI has emerged as a promising technique that significantly reduces scan time while maintaining diagnostic accuracy. Several studies comparing the diagnostic performance of Ab-MRI with that of standard MRI have demonstrated that, despite improving examination efficiency, Ab-MRI shows no significant differences in key diagnostic metrics, including recall rate, cancer detection rate, false-positive biopsy recommendation rate, PPV, SEN and SPE (13, 22). Our study further confirms that Ab-MRI provides comparable diagnostic performance to full-protocol MRI while significantly reducing scan time. However, compared with the reference FDP, the Ab−MRI acquisition protocol exhibits wider confidence intervals, likely due to three inherent limitations. First, the accelerated acquisition reduces temporal resolution, amplifying biological noise arising from intertumoral heterogeneity—especially in DCIS cases with variable necrosis patterns. Second, the lack of a compensation mechanism for dynamic contrast–enhanced phases prevent effective correction of motion artifacts, thereby increasing measurement uncertainty. Third, many low− and intermediate−grade DCIS lesions appear isointense on DWI sequences, which diminishes lesion–to–background contrast and further exacerbates data variability. Although FDP remains the diagnostic gold standard, in a screening context our Ab−MRI protocol—with a clinically acceptable specificity of 85%—demonstrates sufficient reliability and practical value.

By integrating mammographic imaging, MRI features, and pathological data, Ab-MRI allows for accurate prognostic risk assessment in DCIS patients. In addition to offering higher sensitivity and accuracy in imaging evaluation, Ab-MRI enhances diagnostic efficiency, making it a valuable tool for early breast cancer screening and diagnosis.

The novelty of this study lies in the first-ever integration of the Ab-MRI protocol with mammography to stratify the risk of DCIS at different pathological progression stages and explore the potential applications of this technology in breast precancerous lesions. By optimizing MRI sequences, Ab-MRI not only enhances diagnostic efficiency but also provides critical support for precise risk stratification and personalized treatment of DCIS. Despite its clinical value, this study has certain limitations. First, biopsy pathology results indicating intraductal papilloma, borderline phyllodes tumor, or atypical ductal hyperplasia often led to surgical excision in clinical practice. Since final surgical pathology may upgrade these lesions to DCIS or invasive carcinoma, we did not categorize them as benign lesions in our study, which may introduce selection bias. Second, as a single-center retrospective study with a relatively small sample size, the generalizability of our findings may be limited. Furthermore, considering the generally favorable prognosis and long survival of breast cancer patients, this study did not include disease-free survival or overall survival as prognostic endpoints. Lastly, as our study retrospectively analyzed imaging using the Ab-MRI protocol rather than simulating a real-world abbreviated MRI examination, potential bias in the results cannot be excluded.

In conclusion, this study explored the potential of Ab-MRI in the diagnosis of DCIS and utilized the Nottingham grading system for prognostic stratification. By integrating deep learning and natural language processing techniques, along with mammographic and Ab-MRI imaging features, we developed a DCIS prognostic risk prediction model to guide clinical decision-making and support precision medicine.

## Data Availability

The original contributions presented in the study are included in the article/supplementary material. Further inquiries can be directed to the corresponding author.

## References

[1] Siegel RL Kratzer TB Giaquinto AN Sung H Jemal A. Cancer statistics, 2025. CA Cancer J Clin. (2025) 75:10–45. doi: 10.3322/caac.21871 39817679 PMC11745215

[2] Wang J Li B Luo M Huang J Zhang K Zheng S. Progression from ductal carcinoma in *situ* to invasive breast cancer: molecular features and clinical significance. Signal Transduct Target Ther. (2024) 9:83. doi: 10.1038/s41392-024-01779-3 38570490 PMC10991592

[3] Schmitz R van den Belt-Dusebout AW Clements K Ren Y Cresta C Timbres J. Association of DCIS size and margin status with risk of developing breast cancer post-treatment: multinational, pooled cohort study. Bmj. (2023) 383:e076022. doi: 10.1136/bmj-2023-076022 37903527 PMC10614034

[4] Narod SA Iqbal J Giannakeas V Sopik V Sun P. Breast cancer mortality after a diagnosis of ductal carcinoma in situ. JAMA Oncol. (2015) 1:888–96. doi: 10.1001/jamaoncol.2015.2510 26291673

[5] Bleyer A Welch HG. Effect of three decades of screening mammography on breast-cancer incidence. N Engl J Med. (2012) 367:1998–2005. doi: 10.1056/NEJMoa1206809 23171096

[6] Delaloge S Khan SA Wesseling J Whelan T. Ductal carcinoma in *situ* of the breast: finding the balance between overtreatment and undertreatment. Lancet. (2024) 403:2734–46. doi: 10.1016/S0140-6736(24)00425-2 38735296

[7] Rauch GM Kuerer HM Scoggins ME Fox PS Benveniste AP Park YM. Clinicopathologic, mammographic, and sonographic features in 1,187 patients with pure ductal carcinoma in *situ* of the breast by estrogen receptor status. Breast Cancer Res Treat. (2013) 139:639–47. doi: 10.1007/s10549-013-2598-7 PMC3982796 23774990

[8] Lee SH Ryu HS Jang MJ Yi A Ha SM Kim SY. Glandular tissue component and breast cancer risk in mammographically dense breasts at screening breast US. Radiology. (2021) 301:57–65. doi: 10.1148/radiol.2021210367 34282967

[9] Ha SM Jang MJ Youn I Yoen H Ji H Lee SH. Screening outcomes of mammography with AI in dense breasts: A comparative study with supplemental screening US. Radiology. (2024) 312:e233391. doi: 10.1148/radiol.233391 39041940

[10] Patel BK Carnahan MB Northfelt D Anderson K Mazza GL Pizzitola VJ. Prospective study of supplemental screening with contrast-enhanced mammography in women with elevated risk of breast cancer: results of the prevalence round. J Clin Oncol. (2024) 42:3826–36. doi: 10.1200/JCO.22.02819 39058970

[11] Kuhl CK Schrading S Strobel K Schild HH Hilgers RD Bieling HB. Abbreviated breast magnetic resonance imaging (MRI): first postcontrast subtracted images and maximum-intensity projection-a novel approach to breast cancer screening with MRI. J Clin Oncol. (2014) 32:2304–10. doi: 10.1200/JCO.2013.52.5386 24958821

[12] Comstock CE Gatsonis C Newstead GM Snyder BS Gareen IF Bergin JT. Comparison of abbreviated breast MRI vs digital breast tomosynthesis for breast cancer detection among women with dense breasts undergoing screening. Jama. (2020) 323:746–56. doi: 10.1001/jama.2020.0572 PMC7276668 32096852

[13] Jones LI Marshall A Elangovan P Geach R McKeown-Keegan S Vinnicombe S. Evaluating the effectiveness of abbreviated breast MRI (abMRI) interpretation training for mammogram readers: a multi-centre study assessing diagnostic performance, using an enriched dataset. Breast Cancer Res. (2022) 24:55. doi: 10.1186/s13058-022-01549-5 35907862 PMC9338668

[14] Zhang YD Satapathy SC Wu D Guttery DS Górriz JM Wang SH. Improving ductal carcinoma in *situ* classification by convolutional neural network with exponential linear unit and rank-based weighted pooling. Complex Intell Systems. (2021) 7:1295–310. doi: 10.1007/s40747-020-00218-4 PMC8591711 34804768

[15] Narayanan PL Raza SEA Hall AH Marks JR King L West RB. Unmasking the immune microecology of ductal carcinoma in *situ* with deep learning. NPJ Breast Cancer. (2021) 7:19. doi: 10.1038/s41523-020-00205-5 33649333 PMC7921670

[16] Liu C Sun M Arefan D Zuley M Sumkin J Wu S. Deep learning of mammogram images to reduce unnecessary breast biopsies: a preliminary study. Breast Cancer Res. (2024) 26:82. doi: 10.1186/s13058-024-01830-9 38790005 PMC11127450

[17] Fulawka L Blaszczyk J Tabakov M Halon A. Assessment of Ki-67 proliferation index with deep learning in DCIS (ductal carcinoma in *situ*). Sci Rep. (2022) 12:3166. doi: 10.1038/s41598-022-06555-3 35210450 PMC8873444

[18] Huang CY Chang RF Lin CY Hsieh MS Liao PC Wang YJ. Deep-learning model to improve histological grading and predict upstaging of atypical ductal hyperplasia/ductal carcinoma in *situ* on breast biopsy. Histopathology. (2024) 84:983–1002. doi: 10.1111/his.15144 38288642

[19] Alaeikhanehshir S Voets MM van Duijnhoven FH Lips EH Groen EJ van Oirsouw MCJ. Application of deep learning on mammographies to discriminate between low and high-risk DCIS for patient participation in active surveillance trials. Cancer Imaging. (2024) 24:48. doi: 10.1186/s40644-024-00691-x 38576031 PMC10996224

[20] Wu H Jiang Y Tian H Ye X Cui C Shi S. Sonography-based multimodal information platform for identifying the surgical pathology of ductal carcinoma in situ. Comput Methods Prog BioMed. (2024) 245:108039. doi: 10.1016/j.cmpb.2024.108039 38266556

[21] Zhu Z Harowicz M Zhang J Saha A Grimm LJ Hwang ES. Deep learning analysis of breast MRIs for prediction of occult invasive disease in ductal carcinoma in situ. Comput Biol Med. (2019) 115:103498. doi: 10.1016/j.compbiomed.2019.103498 31698241

[22] Lawson MB Partridge SC Hippe DS Rahbar H Lam DL Lee CI. Comparative performance of contrast-enhanced mammography, abbreviated breast MRI, and standard breast MRI for breast cancer screening. Radiology. (2023) 308:e230576. doi: 10.1148/radiol.230576 37581498 PMC10481328

[23] Yang Z Cao Z Zhang Y Tang Y Lin X Ouyang R. MommiNet-v2: Mammographic multi-view mass identification networks. Med Image Anal. (2021) 73:102204. doi: 10.1016/j.media.2021.102204 34399154

[24] Sickles EA. ACR BI-RADS^®^ Atlas, Breast imaging reporting and data system. Am Coll Radiol. (2013) 39.

[25] Goldhirsch A Wood WC Coates AS Gelber RD Thürlimann B Senn HJ. Strategies for subtypes–dealing with the diversity of breast cancer: highlights of the St. Gallen International Expert Consensus on the Primary Therapy of Early Breast Cancer 2011. Ann Oncol. (2011) 22:1736–47. doi: 10.1093/annonc/mdr304 PMC3144634 21709140

[26] Thorat MA Levey PM Jones JL Pinder SE Bundred NJ Fentiman IS. Prognostic value of ER and pgR expression and the impact of multi-clonal expression for recurrence in ductal carcinoma in *situ*: results from the UK/ANZ DCIS trial. Clin Cancer Res. (2021) 27:2861–7. doi: 10.1158/1078-0432.CCR-20-4635 PMC7611296 33727261

[27] Kuerer HM Albarracin CT Yang WT Cardiff RD Brewster AM Symmans WF. Ductal carcinoma in *situ*: state of the science and roadmap to advance the field. J Clin Oncol. (2009) 27:279–88. doi: 10.1200/JCO.2008.18.3103 19064970

[28] Holmes P Lloyd J Chervoneva I Pequinot E Cornfield DB Schwartz GF. Prognostic markers and long-term outcomes in ductal carcinoma in *situ* of the breast treated with excision alone. Cancer. (2011) 117:3650–7. doi: 10.1002/cncr.25942 21319154

[29] Lee AH Ellis IO. The Nottingham prognostic index for invasive carcinoma of the breast. Pathol Oncol Res. (2008) 14:113–5. doi: 10.1007/s12253-008-9067-3 18543079

[30] Lips EH Kumar T Megalios A Visser LL Sheinman M Fortunato A. Genomic analysis defines clonal relationships of ductal carcinoma in *situ* and recurrent invasive breast cancer. Nat Genet. (2022) 54:850–60. doi: 10.1038/s41588-022-01082-3 PMC9197769 35681052

[31] Gorringe KL Hunter SM Pang JM Opeskin K Hill P Rowley SM. Copy number analysis of ductal carcinoma in *situ* with and without recurrence. Mod Pathol. (2015) 28:1174–84. doi: 10.1038/modpathol.2015.75 26321097

[32] de Boniface J Szulkin R Johansson ALV. Survival after breast conservation vs mastectomy adjusted for comorbidity and socioeconomic status: A swedish national 6-year follow-up of 48 986 women. JAMA Surg. (2021) 156:628–37. doi: 10.1001/jamasurg.2021.1438 PMC8100916 33950173

[33] Clarke M Collins R Darby S Davies C Elphinstone P Evans V. Effects of radiotherapy and of differences in the extent of surgery for early breast cancer on local recurrence and 15-year survival: an overview of the randomised trials. Lancet. (2005) 366:2087–106. doi: 10.1016/S0140-6736(05)67887-7 16360786

[34] Monticciolo DL Helvie MA Hendrick RE. Current issues in the overdiagnosis and overtreatment of breast cancer. AJR Am J Roentgenol. (2018) 210:285–91. doi: 10.2214/AJR.17.18629 29091010

[35] Neal CH Joe AI Patterson SK Pujara AC Helvie MA. Digital mammography has persistently increased high-grade and overall DCIS detection without altering upgrade rate. AJR Am J Roentgenol. (2021) 216:912–8. doi: 10.2214/AJR.20.23314 33594910

[36] Chou SS Romanoff J Lehman CD Khan SA Carlos R Badve SS. Preoperative breast MRI for newly diagnosed ductal carcinoma in situ: imaging features and performance in a multicenter setting (ECOG-ACRIN E4112 trial). Radiology. (2021) 301:66–77. doi: 10.1148/radiol.2021219016 34342501 PMC8474971

[37] Grimm LJ Rahbar H Abdelmalak M Hall AH Ryser MD. Ductal carcinoma in situ: state-of-the-art review. Radiology. (2022) 302:246–55. doi: 10.1148/radiol.211839 PMC8805655 34931856

[38] Vag T Baltzer PA Renz DM Pfleiderer SO Gajda M Camara O. Diagnosis of ductal carcinoma in *situ* using contrast-enhanced magnetic resonance mammography compared with conventional mammography. Clin Imaging. (2008) 32:438–42. doi: 10.1016/j.clinimag.2008.05.005 19006771

